# Distribution and Source Apportionment of Polycyclic Aromatic Hydrocarbons (PAHs) in Forest Soils from Urban to Rural Areas in the Pearl River Delta of Southern China

**DOI:** 10.3390/ijerph110302642

**Published:** 2014-03-04

**Authors:** Yihua Xiao, Fuchun Tong, Yuanwen Kuang, Bufeng Chen

**Affiliations:** 1 Research Institute of Tropical Forestry, Chinese Academy of Forestry, Guangzhou 510520, China; E-Mail: zsjcsdwcbf@126.com; 2 College of Forestry, South China Agricultural University, Guangzhou 510642, China; E-Mail: fuchuntong@scau.edu.cn; 3 Key Laboratory of Vegetation Restoration and Management of Degraded Ecosystems, South China Botanical Garden, Chinese Academy of Sciences, Guangzhou 510650, China

**Keywords:** forest soils, Pearl River Delta, polycyclic aromatic hydrocarbons, source apportionment, urban-rural gradient

## Abstract

The upper layer of forest soils (0–20 cm depth) were collected from urban, suburban, and rural areas in the Pearl River Delta of Southern China to estimate the distribution and the possible sources of polycyclic aromatic hydrocarbons (PAHs). Total concentrations of PAHs in the forest soils decreased significantly along the urban–suburban–rural gradient, indicating the influence of anthropogenic emissions on the PAH distribution in forest soils. High and low molecular weight PAHs dominated in the urban and rural forest soils, respectively, implying the difference in emission sources between the areas. The values of PAH isomeric diagnostic ratios indicated that forest soil PAHs were mainly originated from traffic emissions, mixed sources and coal/wood combustion in the urban, suburban and rural areas, respectively. Principal component analysis revealed that traffic emissions, coal burning and residential biomass combustion were the three primary contributors to forest soil PAHs in the Pearl River Delta. Long range transportation of PAHs via atmosphere from urban area might also impact the PAHs distribution in the forest soils of rural area.

## 1. Introduction

Polycyclic aromatic hydrocarbons (PAHs) are a group of organic contaminants which exist ubiquitously in the environment. Environmental PAHs come from two sources: natural processes (e.g., forest fires, volcanic activity, *etc.*) contributing to the background values of PAHs, and anthropogenic activities (e.g., incomplete combustion of fossil fuels, coke production, industrial processes, *etc.*) contributing to the contamination levels of PAHs [1,2,3,4]. Owing to their carcinogenic, mutagenic, and teratogenic effects on organisms, 16 PAHs were specified as priority control target pollutants by the United States Environmental Protection Agency (EPA) [5,6]. Given the increase of widespread extensive concerns about the environmental behavior and the human toxicity of PAHs, global monitoring of the levels and distribution of PAHs in soils is necessary for risk assessment on human health [4,7,8].

Once emitted, PAHs could be widely dispersed in the air, water and could be retained in the soil matrix for a long time after adsorption onto the soil organic matter [9]. Soils and sediments are often the ultimate repository for most of the hydrophobic organic contaminants such as PAHs bonded with particles [10]. It has been well documented that most anthropogenic PAHs will be restricted to the top layer of the soils [11]. 

As the biggest developing country, China has been estimated having annual total PAHs emissions of 25,300 tons [12], contributing over 20% of the global PAH emissions [13]. The emitted PAHs mainly originate from the combustion of fossil fuels and biomass [14,15]. Recently, investigation on PAH concentrations and distribution in atmosphere, water, soils and sediments has been done at different regional scales in China [2,4,8]. As the primary PAH sink in terrestrial ecosystems [12], soils in many areas in China, for example the Pearl River Delta (PRD) [16], the Yangtze River Delta [4,17], and Beijing-Tianjin Area [18], contain the vast majority of PAHs. However, investigation on the concentrations, distribution and possible sources of PAHs in forest soils has been relatively less frequent than in urban and agricultural soils. Compared with common soils, forests soils inevitably have lower PAH levels owing to the effects of the forest canopy. Forest soil is one of the main components of multi-media including atmosphere, plant leaves, soil and runoff [19,20,21]. It has been estimated that forest ecosystems can play vital role in scavenging anthropogenic PAHs [22]. As one of vital components, forest soils act as primary reservoirs of PAHs. The role of forest soils in sequestering PAHs should also be intensively considered. Therefore, the research on the distribution and the possible contributors of PAHs in differently disturbed forest soils was very necessary in assessing forest sequestration and in managing forest risks associated with exposure to these chemicals in soils. 

The environment of the PRD, the most industrialized and urbanized region in southern China, has deteriorated severely during the past decades [23]. Organic contaminants including PAHs, polychlorinated biphenyls, and organochlorine pesticides have been well documented in various environments in this region [8,16,23,24,25]. The objectives of this study were to: (1) Investigate the concentrations and profiles of PAHs in forest soils influenced by urbanization; (2) explore the possible sources of PAHs in the forest soils along an urban–rural gradient in the PRD.

## 2. Materials and Methods

### 2.1. Site Description and Soil Sampling

The PRD includes nine districts covering an area of about 4.2 × 10^4^ km^2^ in Guangdong Province. The annual average temperature is 21–23 °C and ranges from 13–15 °C in January to 28–29 °C in July. The annual precipitation fluctuates between 1,500 and 1,800 mm with about 80% falling in the wet season from April to September [26].

According to previous studies [27], three forest sites representing different disturbances due to urbanization were selected for soil sampling in this study. In order to estimate PAH pollution over the whole region, we selected three moderately polluted sites rather than extreme sites. The urban site was Baiyunshan forest park (23°10′ N, 113°17′ E) in Guangzhou City, the suburban site was Liuxihe National Forest Park (23°32′ N, 113°45′ E) in Chonghua City, and the rural site was Yunjishan Nature Reserve (24°05′ N, 114°08′ E) in Xinfeng City. A total of 45 surface soil (0–20 cm depth) samples from each site were collected from July to August 2013. In order to avoid the influence of point source pollution, sampling spots were chosen away from roads and other emission sources. 

Each sample comprises a mixture of soils containing more than five subsamples within 100 m^2^ and every sample site spacing is more than 50 m. After being transported to the laboratory, these soil samples were air dried, ground to pass through a 2-mm sieve, homogenized, and stored at 4 °C for analysis. 

### 2.2. Chemicals and Materials

The priority 16 PAHs, including naphthalene (NAP), acenaphthene (ACE), acenaphthylene (ACY), fluorine (FLO), phenanthrene (PHE), anthracene (ANT), fluoranthene (FLA), pyrene (PYR), benz[a]anthracene (BaA), chrysene (CHR), benzo[b]fluoranthene (BbF), benzo[k]fluoranthene (BkF), benzo[a]pyrene (BaP), dibenz[a,h]anthracene (DahA), indeno[1,2,3-cd]pyrene (IcdP), and benzo[ghi]perylene (BghiP) (specified by EPA Method 610), in a mixture and a surrogate consisting of naphthalene-d_8_, acenaphthene-d_10_, phenanthrene-d_10_, chrysene-d_12_, and perylene-d_12_, as well as the native standards, were obtained from Ultra Scientific Inc. (North Kingston, RI, USA). Internal standards (hexamethylbenzene) were attained initially as a solid of 99% purity (Aldrich Chemicals, Gillingham, Dorset, UK). The PAH standard reference material (SRM 1491a) was purchased from the National Institute of Standards and Technology (NIST, Gaithersburg, MD, USA). All organic solvents (Nanjing Chemical Reagent Co., Nanjing, China) used in the extraction were redistilled using a glass system. 

### 2.3. Sample Extraction

A 10 g soil sample spiked with 2.5 ng of each deuterated PAHs was mixed with 10 g of anhydrous sodium sulfate and then Soxhlet extracted for 24 h with 200 mL of hexane/acetone (1:1 v/v). The extracts were concentrated to 2 mL by rotary vacuum evaporation and solvent-exchanged to hexane. The concentrated extracts were cleaned by silica gel column chromatography (25 cm × 1 cm i.d). The glass chromatographic column fitted with a Teflon stopcock was packed from the bottom with glass wool followed by 10 g of activated silica and then 2 cm of anhydrous sodium sulfate. After transferring the sample extract, the column was eluted with 25 mL of *n*-hexane and 35 mL of *n*-hexane/DCM (3:2, v/v), respectively. The first fraction containing *n*-alkanes was discarded and the second fraction containing PAHs was collected. Afterward, the collected PAHs fraction was vacuum-evaporated, solvent exchanged to isooctane, and then concentrated to 0.2 mL under a gentle stream of nitrogen before GC/MS analysis.

### 2.4. GC Analysis and Quantification

The determination of PAHs was performed on an Agilent 6890 N gas chromatograph-5973 mass selective detector (GC-MSD) system (Agilent Technologies Inc., Santa Clara, CA, USA) equipped with a fused silica capillary HP-5MS column (30 m × 0.25 mm × 0.25 mm). The carrier gas was helium at a constant flow rate of 1.5 mL∙min^−1^. The column temperature was programmed to rise from 70 to 200 °C at 3 °C∙min^−1^ and then to 285 °C at 5 °C∙min^−1^ and held for 12 min at 280 °C. A 1 μL sample was injected in splitless mode. The interface, ion source, and quadrupole temperatures were maintained at 280, 150, and 160 °C, respectively. The ionization was carried out in the electron impact mode at 70 eV and the data were acquired under selected ion monitoring (SIM) mode. Identification of PAHs was based on the selected ions and the comparison of retention time between samples and the standard solution containing individual PAHs. The quantitation of PAH was done by using relative response factor of target PAHs to internal standards. For target PAHs, one or more quantitation ions and confirmation ions were used.

### 2.5. Quality Control

The instrument was calibrated daily with calibration standards and the relative percentage difference between the four-point calibration and daily calibration was less than 20%. Method blanks (solvent), spiked blanks (standards spiked into the solvent), sample duplicates, and a sample from the National Institute of Standards and Technology standard reference material (NIST 1941b) were analyzed routinely with field samples. In addition, the surrogate standards were added to all the samples (including quality assessment samples) to monitor procedural performance. The surrogate recoveries (%) in field samples were as follows: naphthalene-d_8_ = 47.2 ± 7.5; acenaphthene-d_10_ = 81.3 ± 4.4; phenanthrene-d_10_ = 96.3 ± 3.9; chrysene-d_12_ = 92.8 ± 6.7; and perylene-d_12_ = 96.6 ± 5.8.The method detect limit (MDL) was calculated to be in the range of 0.6–2.6 ng∙g^−^^1^ DW using the Method Detection Limit Calculator [28]. The PAH concentrations in this study are presented on a dry weight basis. 

### 2.6. Statistical Analysis

Statistical analyses including one-way analysis of variance (ANOVA), correlation analysis, and principal component analysis (PCA) were performed using SPSS 17.0 for Windows (SPSS Inc., Chicago, IL, USA). The concentrations of PAHs were log-transformed to achieve normal distribution prior to the statistical analysis.

## 3. Results and Discussion

### 3.1. PAH Concentrations

Concentrations of total PAHs (ΣPAHs) in the 45 forest soil samples from the urban, suburban, and rural areas ranged from 71.28 to 515.34, 39.85 to 201.01, and 18.90 to 75.17 ng∙g^−1^, respectively (Table 1). As expected, PAHs concentrations showed a strong urban–suburban–rural gradient. The distribution pattern of ΣPAHs indicated a significant influence of urbanization on forest soils. According to the results shown in Table 1, the highest ΣPAHs was found in the urban forest area (152.10 ± 79.41 ng∙g^−1^, mean ± standard deviation (S.D.), as follows), and is comparable to the corresponding levels in the urban forest soils of Klang Valley, Malaysia (155.20 ± 91.40 ng∙g^−1^) [29]. Compared to other soils in the PRD, e.g., the paddy fields (253.00 ± 130.00 ng∙g^−1^) [8], vegetable soils (318.20 ± 156.40 ng∙g^−1^) [25], crop soils (218.00 ± 123.00 ng∙g^−1^), and particularly the urban soils (960.00~1,800.00 ng∙g^−1^) [16,30], the ΣPAHs in urban forest soils in this study was significantly lower. The mean concentrations of ΣPAHs in suburban forest soils (73.67 ± 16.34 ng∙g^−1^) was about half of those in the urban area and double those in the rural area. Compared to suburban residential areas in the PRD, such as Guangzhou (160.00~1,300.00 ng∙g^−1^), Dongguan (128.00~357.00 ng∙g^−1^), and Shenzhen (230.00~3,700.00 ng∙g^−1^) [31,32], and agricultural soils in suburban areas around Guangzhou (422.00 ± 194.35 ng∙g^−1^), PAH concentrations in suburban forest soils were much lower. This comparison implied that urban forests could play important role in scavenging PAHs from the atmospheric deposition, which was consistent with the findings by Tian *et al.* (2008) [33]. The concentrations of PAHs in the rural forest soils (35.86 ± 6.91 ng∙g^−1^) were similar to the rural areas around the world, for example, volcanic mountains in the subtropical Atlantic (33.70 ng∙g^−1^) [34], tropical regions like Ghana (31.20 ng∙g^−1^) [35], mountains of Western Canada (30.00 ng∙g^−1^) [36], and mountains of Pohang South Korea (25.60 ng∙g^−1^) [37].

**Table 1:** Concentrations (ng∙g^−1^ dry weight) of PAHs in forest soils in urban, suburban, and rural areas.

PAH	Urban			Suburban			Rural		
	Average	Min	Max	Average	Min	Max	Average	Min	Max
NAP	20.42	7.18	34.04	19.20	10.41	29.29	13.77	8.59	22.87
ACY	0.94	0.30	1.57	0.71	0.43	1.33	0.30	0.13	0.49
ACE	0.92	0.18	4.10	0.86	0.54	1.39	0.41	0.25	0.53
FLO	1.50	0.56	3.05	2.94	1.69	5.20	1.69	0.98	2.66
PHE	14.05	0.66	30.98	15.48	6.51	41.04	7.01	3.87	13.57
ANT	5.87	2.20	61.72	2.46	1.29	5.04	2.32	1.35	4.69
FLA	22.77	7.86	94.72	7.27	2.31	32.97	2.01	1.05	4.49
PYR	20.09	9.94	43.22	5.72	2.30	16.23	1.24	0.74	2.12
BaA	6.54	1.98	18.72	2.41	0.88	6.30	1.07	0.42	3.69
CHR	11.81	3.65	41.81	4.00	1.27	11.18	1.79	0.66	7.30
BbF	13.44	2.25	69.87	4.80	0.94	12.44	1.85	0.43	11.40
BkF	10.07	1.09	69.33	1.27	0.22	3.57	0.33	0.10	1.49
BaP	4.59	0.95	29.67	1.14	0.19	2.74	0.27	0.07	0.74
IcdP	9.32	1.21	66.43	2.82	0.68	9.36	0.94	0.28	3.29
DahA	2.96	0.23	11.01	0.45	0.10	0.98	0.16	0.04	1.06
BghiP	6.84	1.46	23.76	2.15	0.39	4.80	0.72	0.21	4.39
ΣPAHs ^a^	152.10	71.28	515.34	73.67	39.85	201.01	35.86	18.90	75.17
PAHs7 ^b^	58.72	13.63	279.47	16.88	4.88	51.93	6.41	2.11	28.72
PAHs7c/PAHs	0.39	0.19	0.54	0.23	0.12	0.26	0.18	0.11	0.38
R3 ≤ /R ≥ 4 ^c^	0.41	0.14	1.53	1.30	0.41	2.33	2.46	0.38	3.34

Note: ^a^ Total concentrations of 16 individual PAHs; ^b^ ∑PAH_7c_: concentrations of seven carcinogenic PAHs; ^c^ Ratio of sum concentration of low molecular weight PAHs (≤3 rings) to that of high molecular weight PAHs (≥4 rings).

According to the classification system suggested by Maliszewska-Kordybach [38], a soil concentration of 200–600 ng∙g^−1^ PAHs represents weak contamination and a concentration below 200 ng∙g^−1^ indicates no contamination. Compared with the results of other studies, the ΣPAHs of forest soils in this research fell within the range of low to middle levels. However, the concentrations of ΣPAHs in the rural area were much higher than those of endogenous sources (1–10 ng∙g^−1^) resulting from plant synthesis and natural fires [39], implying that anthropogenic PAHs from urban area might also contribute to the forest soils via atmospheric long-range transportation. None of the PAH concentrations of soil samples in this study were more than 600 ng∙g^−1^, and 17.8 and 4.4% of the samples in urban and suburban forest soils, respectively, were weakly contaminated. 

### 3.2. PAH Profiles

**Figure 1:**
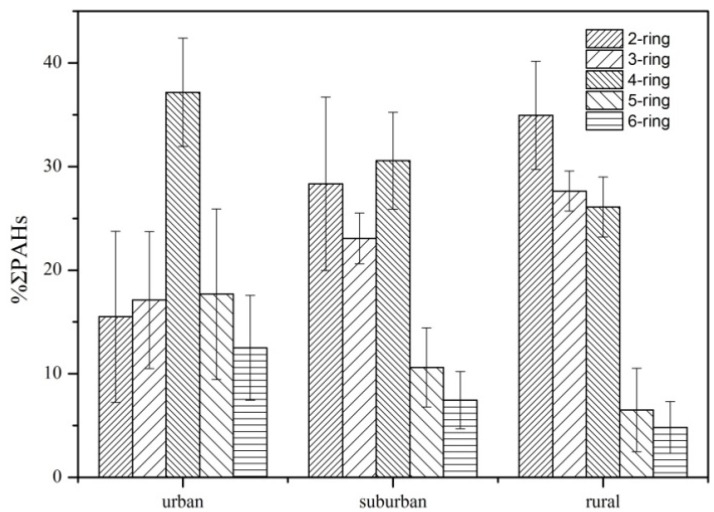
Composition profile of PAHs in forest soils.

The composition profiles of PAHs in the three areas are presented in Figure 1. The profile of high molecular weight PAHs (HMW-PAHs) decreased along urban–suburban–rural transect, e.g., 6-ringed PAHs comprised from 12.5% of ΣPAHs in the urban areas to 4.8% in the rural areas, whereas the fractions of low molecular weight PAHs (LMW-PAHs, including 2- and 3-ringed PAHs) increased along the gradient with 2-ringed PAHs from 13.5 to 39.0% and 3-ringed PAHs from 15.1 to 32.6%. 

Figure 1 also showed that HMW-PAHs dominated in the urban forest soils while LMW-PAHs dominated in suburban and rural soils. Interestingly, the dominance of 2, 3-ringed PAHs was also found in less polluted soils such as tropical [39,40] and mountain soils [36], whereas 4-6-ringed PAHs dominated the composition in urban soils [41,42,43,44,45]. Additionally, the composition and the relative abundance of individual PAHs varied considerably. 

**Figure 2:**
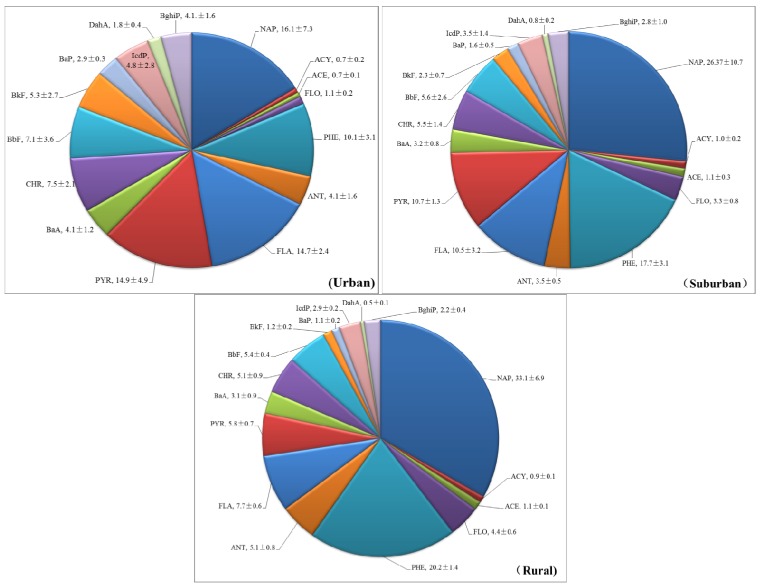
Contribution of the individual PAH compounds to the total PAHs in forest soils along the urban–suburban–rural transect (%).

Figure 2 shows that the percentages of individual PAHs to ΣPAHs in our soil samples. NAP was the most dominant component, representing 16.1%, 26.4%, and 33.1% of ΣPAHs in urban, suburban, and rural sites, respectively. NAP, PHE, ANT, FLA, and PYR were dominant components, which account for 55.8% and 65.3% to ΣPAHs in urban and suburban sites, respectively. NAP and PHE were dominant components in rural site with 53.3% of percentage to ΣPAHs.

Emission sources as well as long range transportation of PAHs via atmosphere contribute greatly the composition profiles of environmental PAHs [46]. The urban–suburban–rural gradient of composition profiles might be ascribed to different emissions. As well known, LMW-PAHs were chiefly generated by low- or moderate-temperature combustion process (such as biomass combustion and domestic coal burning) while HMW-PAHs were mainly generated by high-temperature combustion process (such as vehicular exhausts and industrial coal combustion). In the urban areas, there were usually much devise of emissions (traffic, industrial, *etc.*) than in the rural ones. The larger fraction of LMW-PAHs in the rural forest soils in this study indicated that PAHs might mainly stem from low- or moderate-temperature combustion processes. The higher fraction of HMW-PAHs (49.1%–74.7%) in the urban forest soils implied that there were additional industrial emissions contributing to soil PAHs. As demonstrated by numerous researchers, traffic and industrial emissions were the main contributors to atmospheric PAHs in particulates at the urban centers and at the locations close to major highways [47,48,49], so we inferred that industrial and traffic emissions have caused significant accumulation of PAHs in the urban forest soils*,* while atmospheric transportation and deposition of pyrogenic–stemmed PAHs also contributed to the forest soils PAHs at rural areas*.* Some PAHs, such as Nap, might be emitted by biogenic sources, in particular at forest sites [20], which partly explained the dominance of LMW-PAHs at the rural site (Figure 1 and Figure 2). Furthermore, in rural areas, coal burning and biomass combustion are the major sources of PAH contamination, and the emission factors for low-ringed PAHs from biomass burning are higher than those for coal combustion [14]. Additionally, there is more photochemical degradation of LMW PAHs in urban areas than in rural villages because of OH radicals [50], which further explained why higher concentrations of 2,3-ringed PAHs existed in the rural village area than in the urban area. The urban–suburban–rural gradient obtained in this research verified the differences in emission sources. 

### 3.3. Source Apportionment by Isomeric Ratios of PAHs

The anthropogenic release of PAHs can be attributed to petrogenic and pyrogenic origins. The PAHs of petrogenic origins are characterized by the predominance of 2- and 3-ringed PAHs, while the PAHs from pyrogenic origins are characterized by a high proportion of PAHs with more than four rings. Beside the ratio of LMW- and HMW-PAHs to total PAHs, several PAH congener ratios, such as Ant/(Phe + Ant), Fla/(Fla + Pyr), Phe/(Phe + Ant), BaA/(BaA + Chr), Flu/(Flu + Pyr), and InP/(InP + BP), have been widely used to distinguish the possible source categories of environmental PAHs [51,52,53]. 

A ratio of Ant/(Ant + Phe) < 0.1 is indicative of a petroleum source, while a ratio > 0.1 is indicative of combustion. Meanwhile, a ratio of Fla/(Fla + Pyr) lower than 0.40 indicates a petroleum source and one higher than 0.50 indicates a biomass and coal combustion sources, while a ratio between 0.4 and 0.5 is characteristic of liquid fossil fuel combustion. A ratio of IcdP/(IcdP + BghiP) lower than 0.20 indicates a petroleum source, one higher than 0.50 indicates biomass and coal combustion sources, and one between 0.20 and 0.50 indicates liquid fossil fuel combustion [51]. Moreover, a ratio of BaA/(BaA + Chr) less than 0.2 suggests a petroleum source, 0.2–0.35 suggests petroleum combustion (especially liquid fossil fuel, vehicle and crude oil), and a ratio greater than 0.35 suggests combustion of coal, grass, and wood [54,55]. 

**Figure 3:**
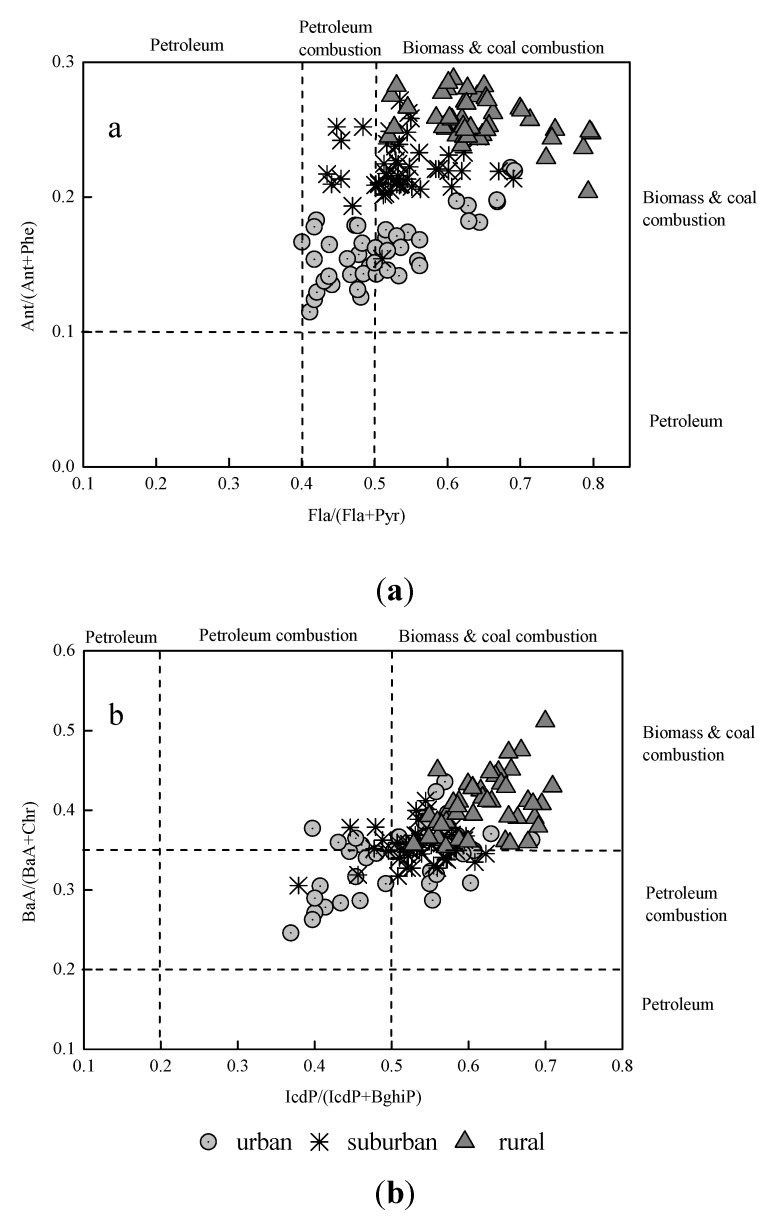
Cross-plot of the isomeric ratios of: (**a**) Ant/(Ant+Phe) *vs.* Flu/(Flu + Pyr), and (**b**) BaA/(BaA + Chr) *vs.* InP/(InP + BP) in forest soil in urban, suburban, and rural sites.

In the present study, the ratios of **A**nt/(Ant + Phe) varies between 0.11 and 0.29 in forest soils, and the ratios of Fla/(Fla + Pyr) were higher than 0.4. In addition, 48.9 and 15.6% of the ratios of Fla/(Fla + Pyr) were 0.4–0.5 in the urban and suburban forest soils, respectively (Figure 3a). However, the ratios of Fla/(Fla + Pyr) were more than 0.5 in rural forest soils. The IcdP/(IcdP + BghiP) ratios changed from 0.37 to 0.71 in all of the forest samples, and samples from rural forest soils had ratios above 0.5. Furthermore, 60.0 and 84.4% of the IcdP/(IcdP + BghiP) ratios were above 0.50 in urban and suburban forest soils, respectively, while 40.0 and 15.6% of the IcdP/(IcdP + BghiP) ratios varied between 0.2 and 0.5 in urban and suburban forest soils, respectively. The ratios of BaA/(BaA + Chr) varied from 0.28 to 0.51, with most of them higher than 0.35 (Figure 3b). The above ratio values implied that traffic emission and coal combustion might contribute to the occurrence of PAHs in urban forest soils, while biomass- and coal-related residential heating might contribute to the forest soil PAHs at rural area. The results were similar to previous reports from the urban soils in the PRD [25,32,56,57].

### 3.4. Source Identification by Principal Component Analysis

Principal component analysis (PCA) was conducted to reduce the set of original variables and to extract a small number of latent factors for analyzing the relationship among the observed variables for further investigations of possible sources of PAHs. Two principal components (PC1and PC2) were extracted with eigenvalues > 1, accounting for the majority (>85%) of the total variances (Table 2) at the three forest soils.

As well known, LMW-PAHs such as PHE, ANT, and FLO are indicative of coke oven origin [58], NAP accounts for the majority of the mass in coke oven, highway tunnel, and gasoline engine samples [59], and ACY and ACE are markers for domestic wood combustion [60]. On the other hand, HMW-PAHs such as FLA, PYR, BaA, CHR, BbF, BkF, BaP, IcdP, DahA, and BghiP are typical markers for pyrolysis or incomplete combustion. FLA, PYR, BaA, CHR, and BaP are markers for coal combustion [56,57,58]. BbF and BkF are components of fossil fuels and portions of them are associated with their combustion [61]. BaA and CHR often result from the combustion of both diesel and natural gas [59,60]. IcdP, BghiP, and DahA are associated with traffic emission [60]. 

**Table 2:** Principal component analysis after Varimax rotation for the selected PAHs in forest soils in urban, suburban, and rural sites. Only factors with loading values greater than 0.5 were listed.

Site	Urban		Suburban		Rural	
Principal component	PC1	PC2	PC1	PC2	PC1	PC2
NAP		0.86	0.91		0.82	
ACY		0.91	0.90		0.94	
ACE		0.88	0.81		0.98	
FLO		0.53	0.73	0.67	0.92	
PHE		0.57	0.69	0.74	0.70	0.61
ANT		0.66	0.89		0.92	
FLA	0.77		0.72		0.84	
PYR	0.87		0.73			0.75
BaA	0.64		0.95			0.64
CHR	0.68		0.92			0.68
BbF	0.77			0.98		0.71
BkF	0.79			0.97		0.57
BaP	0.89		0.86			
IcdP	0.82			0.88		
DahA	0.86			0.97		
BghiP	0.98			0.84		
Eigenvalues	12.44	1.09	13.35	1.20	10.03	3.73
Variance (%)	79.49	8.10	63.41	27.47	72.67	13.32
Pollution source	Traffic emission	Coal combustion	Coal and biomass combustion	Traffic emission	Biomasscom bustion	Coal combustion

PC1 explained 79.49, 63.41, and 72.67% of total variances in forest soils in urban, suburban, and rural sites, respectively (Table 2). It was characterized by high loadings of 4-6-ringed PAHs, including BaP, IcdP, DahA, BghiP, and PYR, and moderate loadings of FLA, BaA, CHR, BbF, and BkF in urban forest soils. Suburban forest soils were characterized by loadings of 3-6-ringed PAHs, including NAP, ACY, ACE, ANT, FLA, PYR, BaA, and BaP, and moderate loadings of FLO, PHE, FLA and PYR. Rural forest soils were characterized by main loadings of 2,3- and 6-ringed PAHs, including NAP, ACE, FLO, PHE,ANT, FLA, IcdP, DahA, and BghiP. Consequently, PC1 reflected a pyrogenic source (traffic emission) of PAHs in the urban forest soils. The origin of PAHs in suburban forest soils can be attributed to a pyrogenic (coal combustion) source, while the PAHs in rural forest soils mainly originated from wood and coal combustion sources.

PC2 accounted for 8.10, 27.47, and 13.32% of total variances in urban, suburban, and rural sites, respectively (Table 2). It was dominated by LMW-PAHs, such as NAP, ACY, ACE, FLO, PHE, and ANT in urban soils, indicating mixed sources of petroleum and low temperature combustion in those areas. Suburban sites were dominated by HMW-PAHs, such as FLO, PHE, BbF, BkF, IcdP, DahA, and BghiP, which indicated traffic emission and coke oven origins. In contrast, rural forest soils were dominated by HMW-PAHs such as PYR, BaA, CHR, BbF, and BkF, indicating that coal combustion may be the source of PAHs in rural sites. 

These results are also in agreement with previous observations by Cai *et al.* and Liu *et al.* in urban soils [8,16]. Mixed sources (traffic emission, biomass and coal combustion) contributed to the suburban sites, and residential emissions were the dominant source at rural sites [43,62], which is in accordance with our results. 

## 4. Conclusions

Concentrations of PAHs in forest soils showed a strong urban–suburban–rural gradient, with total concentrations up to four times higher in urban areas than in rural ones. HMW-PAHs and LMW-PAHs dominated in the urban forest soils and rural forest soils, respectively. The composition profiles of PAHs displayed an urban–suburban–rural gradient owing to the emission sources, implying the influence of urbanization on the distribution of PAHs in forest soils. The isomeric diagnostic ratios of PAHs revealed that forest soil PAHs were mainly originated from traffic emission, mixed sources and coal/wood combustion in the urban, suburban and rural areas, respectively. Traffic emission, coal combustion and residential biomass emission were the three primary contributors to forest soil PAHs in the PRD. Forest soils in the urban and suburban areas of PRD were weakly contaminated by PAHs. Long range transportation via atmosphere from urban area can also impact forest soils in rural area.
